# A Survey of Knowledge and Barriers of Healthcare Professionals toward Opioid Analgesics in Cancer Pain Management

**DOI:** 10.1155/2022/1136430

**Published:** 2022-04-12

**Authors:** Nehad M. Ayoub, Malak Jibreel, Khawla Nuseir, Ghaith M. Al-Taani

**Affiliations:** ^1^Department of Clinical Pharmacy Faculty of Pharmacy, Jordan University of Science and Technology, Irbid 22110, Jordan; ^2^Department of Clinical Pharmacy and Pharmacy Practice, Faculty of Pharmacy, Yarmouk University, Irbid, Jordan

## Abstract

**Purpose:**

Pain is among the most frequent and troubling symptoms in cancer patients. Despite the availability of updated treatment guidelines and effective pharmacological therapies, undertreatment of cancer pain remains a global problem. Opioids are the mainstay analgesics to treat moderate-to-severe cancer pain. The goal of this study was to assess the knowledge and barriers towards opioid analgesics for cancer pain management among healthcare professionals in Oncology Units in Jordan.

**Methods:**

A structured questionnaire was administered to healthcare professionals (consultant doctors, resident doctors, pharmacists, and nurses) at three Oncology Units in a cross-sectional study design.

**Results:**

A total of 201 healthcare professionals completed the questionnaire. The average age was 34.8 ± 8.1 years (range 23–58) and 49.3% of respondents were nurses. The mean score for the knowledge of opioids was 12.5 ± 3.2 out of 24 points (range 2–20). An acceptable level of knowledge was observed in 50.7% of participants, while 49.3% had poor knowledge. Knowledge items mostly answered incorrectly were related to opioid administration, pharmacology, dosing, adverse events, rotation, and toxicity. Knowledge scores were significantly higher for consultant doctors compared to pharmacists and nurses (*p*=0.016 and *p* < 0.001, respectively). Healthcare professionals who handled opioid analgesics had significantly higher mean knowledge scores than those who did not (*p*=0.012). Linear regression analysis revealed that being a consultant physician has an independent, statistically significant association with higher knowledge scores. Among perceived barriers to using opioids, fear of addiction by patients was the most frequently reported barrier by respondents (79.6%). Other highly recognized barriers were fear of adverse effects by patients (67.2%) and lack of training programs on opioid dosing and monitoring (63.7%).

**Conclusions:**

This study revealed major gaps in the knowledge of opioids and pain management among healthcare professionals. There is an urgent need for developing innovative interventions to improve the knowledge of opioid analgesics and the understanding of pain management guidelines among healthcare professionals in Jordan.

## 1. Introduction

Pain is a frequent and extremely troublesome symptom in cancer patients [1]. The prevalence of pain ranges from 14% to 100% among cancer patients and increases in the advanced stages of the disease [2]. According to a systematic review and meta-analysis, pain was reported by 55% of cancer patients during anticancer treatment and by 66.4% in advanced, metastatic, or terminal disease [3]. Further, pain intensity was moderate to severe in 38% of patients. Pain complicates the clinical condition, causes emotional distress, and adversely affects the quality of life of cancer patients [3–5]. Accordingly, adequate management of cancer pain is of paramount importance for patients with cancer and a cornerstone of comprehensive cancer care [2].

Cancer pain is characterized by its molecular complexity rendering its pharmacologic management challenging. The etiology of cancer pain is multifactorial, and it may involve somatic, visceral, neuropathic, and inflammatory mechanisms [6]. Cancer pain may present as an acute pain that has a quick onset and usually lasts less than 3 to 6 months or as a chronic pain that is persistent and lasts more than 3 to 6 months [7]. In patients with well-controlled background pain, breakthrough cancer pain is a transient exacerbation of severe pain state that negatively impacts the quality of life of patients [7, 8]. Several guidelines are available for the management of cancer pain including the World Health Organization (WHO), the National Comprehensive Cancer Network (NCCN), [9] the European Association for Palliative Care (EAPC), [10] the European Society for Medical Oncology (ESMO), [11] and the American Society of Clinical Oncology (ASCO) clinical practice guidelines [12]. Among these, the WHO three-step ladder is a globally recognized guide for cancer pain management [13]. According to the WHO recommendations, opioid narcotics should be considered as the analgesic therapy of choice for patients with moderate-to-severe cancer pain. The WHO standards also recommend the use of adjuvants in conjunction with opioids in the treatment of cancer pain. Adjuvants are drugs with other indications but have an analgesic effect and are commonly used in current clinical practice for the treatment of cancer pain [14]. This includes the use of steroids, antidepressants, and anticonvulsants as adjuvant medicines.

Opioids are a group of analgesic agents commonly used in clinical practice [15]. Opioids are naturally derived from the opium poppy or synthesized to act on opioid receptors in the central nervous system to produce analgesia [15]. Opioids can be classified according to their effect on target opioid receptors into agonists, partial agonists, and antagonists. Opioids with agonist activity bind to mu-opioid receptor (MOR) leading to favorable analgesic activity. Besides analgesia, the activation of MOR by opioids causes several adverse effects including sedation, nausea and vomiting, constipation, bradycardia, and respiratory depression [16]. The administration of prophylactic treatments concomitantly with opioids to alleviate the most common side effects such as nausea and vomiting and constipation is recommended in current clinical settings. In the management of cancer pain, an opioid analgesic may be changed into another opioid in case a therapeutic effect was lacking or toxicity has developed. This switching from one opioid analgesic to another for a therapeutic purpose is known as opioid rotation [17]. Morphine is considered the archetypical opioid analgesic and remains the gold standard treatment for cancer pain management [15, 17].

Despite the availability of opioid analgesics and updated treatment guidelines, cancer pain remains largely undertreated and patients with moderate-to-severe pain do not receive adequate treatment [17, 18]. Inadequate pain management can be attributed to barriers related to healthcare professionals, patients, and the healthcare system [18–20]. Earlier studies showed that healthcare professionals are insufficiently trained about opioid analgesics and lack knowledge regarding opioid administration and dosages [20, 21]. Opiophobia which is a term characterizing prejudice against the use of opioid analgesics because of exaggerated concerns has been also encountered among healthcare professionals [22]. Such concerns are related to fear of the addictive potential of opioids and the risk of respiratory depression [20]. In Jordan, undertreatment of cancer pain has been a constant problem that did not improve with time [23, 24]. We recently reported that 80.3% of cancer patients had a negative pain management index score, meaning that the prescribed analgesics were inadequate to control pain in these patients [23]. A minority of cancer patients (15.7%) received an opioid analgesic to control pain and the use of nonopioid adjuvants was minimal (21.4%) [23]. In Jordan, few studies were conducted to assess knowledge of cancer pain and its management among healthcare professionals [25]. Considering the continuing problem of inadequate pain management among Jordanian cancer patients along with the fact that opioid analgesics are the mainstay therapy to treat cancer pain, this study was conducted to evaluate the knowledge of and barriers toward the use of opioid analgesics for cancer pain management among oncology healthcare professionals by surveying medical oncologists, pharmacists, and nurses in three Oncology Units in Jordan. The study also aimed to explore the associations between demographic and practice variables and the level of knowledge.

## 2. Methods

### 2.1. Study Design and Settings

A descriptive cross-sectional research design was applied to this study. At present, there are three Oncology Units in Jordan where cancer care is provided [25], public, educational, and military. The study protocol and procedure were approved by the Institutional Review Board committees of Jordan University of Science and Technology (JUST) (research number 44/141/2021), the Ministry of Health, and the Research Ethics Committee of the Royal Medical Services.

### 2.2. Study Population

The study included healthcare professionals (oncologists, pharmacists, and nurses) who work at the Oncology Units and are actively involved in cancer patient management. Healthcare professionals who have a Bachelor's degree in their specialty or a higher degree and an experience of not less than 2 months at the Oncology Unit were eligible to participate in this study. Assistant nurses and pharmacy technicians who have a diploma were excluded from this analysis.

### 2.3. Data Collection Form and Scoring

A structured, self-administered questionnaire was used to collect responses. The questionnaire was developed and modified by the researchers based on previous literature and was administered to participants in the English language [18, 21, 26–29].

The questionnaire face and content validity were evaluated by five faculty members at the Faculty of Pharmacy at JUST in the related field to assess the comprehensiveness, appropriateness, use of terms, the organization, and the accuracy of the study tool. The questionnaire was then examined by an expert group of eight specialists including medical doctors, nurses, and clinical pharmacists who were asked to fill in the questionnaire and give their opinion and comments. Relevance and clarity of the survey questions were further evaluated through a pilot study (*n* = 10). Feedback and comments by the pilot group resulted in minor edits to the survey tool, which was considered to improve the clarity and understanding of the survey items. Data from the pilot sample were excluded from the final analysis. The questionnaire included three parts to be completed in 10–15 min. The first part described demographics and practice settings and had 15 items including age, gender, specialty, hospital, awareness of the WHO ladder, handling opioids, awareness of adjuvant drugs, and others.

The second part included a 24-item scale (20 True/False and 4 Multiple-Choice questions) to assess the knowledge of opioids and their use for cancer pain management. This part includes questions on the principles of using opioids in cancer pain management, pharmacology of opioids, opioid dosing, equianalgesic dosing, administration, titration, rotation, adverse effects, toxicity/overdose, and opioid abuse. The statements listed are based on previously published studies and other literature relevant to opioids and the guidelines provided by the WHO ladder [13, 16]. Responses ranged between “True,” “False,” and “I do not know” for the True/False questions. For questions in the Multiple-Choice format, four choices were provided (tolerance, dependence, addiction, and chemical coping). Thereafter, each correct response was scored 1 point and each of the incorrect and I do not know responses was scored 0 points. The total score ranged from 0 to 24 points on the knowledge of opioids. Healthcare professionals who scored 0–12 points were considered to have poor knowledge, while those with 13 points or more were determined to have acceptable knowledge of opioid analgesics.

The third part describes perceived barriers towards the utilization of opioid analgesics for cancer pain management. 21 potential barriers were listed and divided into those related to medical staff (8 items), barriers related to the healthcare system (8 items), and patient-related barriers (5 items). In this study, the internal consistency reliability coefficients (Cronbach's *α*) calculated for knowledge and barrier items were 0.730 and 0.740, respectively.

### 2.4. Data Collection Procedure

All healthcare professionals in the Oncology Units of the three participating hospitals were recruited to this study. Eligible participants were approached by a trained research assistant who explained the study purpose and procedure. Signed informed consents were obtained from healthcare professionals who agreed to complete the survey questions and were handed out the questionnaire and requested to fill it in while the research assistant was available. This approach allows for consistency in dealing with any issues raised during data collection and improves response rate.

### 2.5. Statistical Analysis

Data analysis was performed using IBM SPSS statistical package (IBM Corp. Version 23.0. Armonk, NY, USA). Descriptive statistics were used to report study variables. Continuous variables are presented as mean ± standard deviation (SD), and categorical variables are presented as frequency and percentages (*n*, (%)). Differences between groups were determined by independent Student's *t*-test for two-group comparisons or one-way analysis of variance (ANOVA) followed by Tukey's HSD post hoc test for multiple group comparisons. Pearson's bivariate correlation analysis was performed to test for correlations between continuous variables. Association between demographic variables and total knowledge scores was assessed using a multivariable linear regression model. Initially, variables that were assumed to be associated with the outcome variable were fitted into a univariable linear regression model. The candidate variables to be included in the model were those with *p* value <0.250 to allow the inclusion of variables with a trend of association. Those candidate variables were then included in an automatic linear regression model that retained important variables that explained the outcome variable. All *p* values were two-sided, and differences were statistically significant at *p* < 0.05.

## 3. Results

Data collection took place over 4 months from September 2020 to December 2020. All eligible healthcare professionals available at the time of data collection were invited to participate in the study (*n* = 250) and 201 completed the questionnaire generating a response rate of 80.4%.

### 3.1. Demographics and Practice Characteristics

Table 1 shows the demographic and practice characteristics of healthcare professionals from three Oncology Units. The average age was 34.8 ± 8.1 years (range 23–58), and most were females (56.7%). Almost half of the participants were nurses (49.3%). Experience in Oncology Unit ranged from 2 months to 27 years (mean ± SD: 5.5 ± 4.9 years). 99 participants (49.3%) had previous education or training in cancer pain management. Less than half of participants indicated that cancer pain is managed by a multidisciplinary team and that specific protocols are applied in management (47.3% and 36.8%, respectively). 121 participants (60.2%) are aware of the WHO pain ladder. The vast majority of healthcare professionals (91.5%) handled opioids in their practice settings. Opioids most commonly encountered were morphine (93.5%), tramadol (91.5%), and fentanyl (61.2%). Regarding the use of adjuvant drugs, 178 participants (88.6%) were aware of them, and steroids were the most common type of adjuvant therapy recognized by participants (79.6%). Other demographic and practice data are shown in Table 1.

### 3.2. Knowledge of Healthcare Professionals regarding Opioids in Cancer Pain Management

Table 2 summarizes the responses reported by participants through descriptive statistics. The mean score for the knowledge of opioids was 12.5 ± 3.2 (range 2–20). 102 participants (50.7%) were determined to have an acceptable level of knowledge, while 99 participants (49.3%) had poor knowledge. Our results showed that the most correctly answered questions were about the indication to using opioids to treat patients with moderate-to-severe cancer pain (91.0%, item 1), the indication of opioids in the management of breakthrough cancer pain (81.6%, item 5), and the use of naloxone to stop opioid overdose (82.6%, item 20). Responses regarding pharmacology, dosing, titration, and administration of opioids were incorrectly answered by most healthcare professionals in this survey. Regarding the pharmacology of opioids, only 39.3% of respondents answered correctly (item 6). Almost one-third of participants correctly answered statements on the equianalgesic dosing and titration for opioids (items 10 and 11). The indication to start laxatives with opioids was correctly answered by 76.6% (item 14). Interestingly, a small proportion of participants (16.9%) were able to indicate that “Opioid analgesics have a high risk of addiction” is a false statement, while 81.6% of participants responded incorrectly to this statement (item 16). Other questions frequently answered incorrectly included those regarding the risk of respiratory depression upon using therapeutic doses of opioids (36.8%, item 17) and the classic triad for opioid overdose (22.4%, item 19). The definitions of tolerance and dependence were the most identified by respondents (78.1% and 59.2%, respectively, items 21 and 22). However, the term “chemical coping” was the least recognized by participants (39.8%, item 24). Responses to other questions are shown in Table 2.

### 3.3. The Impact of Demographic and Practice Characteristics on Knowledge of Opioids among Healthcare Professionals

**Figure 1:**
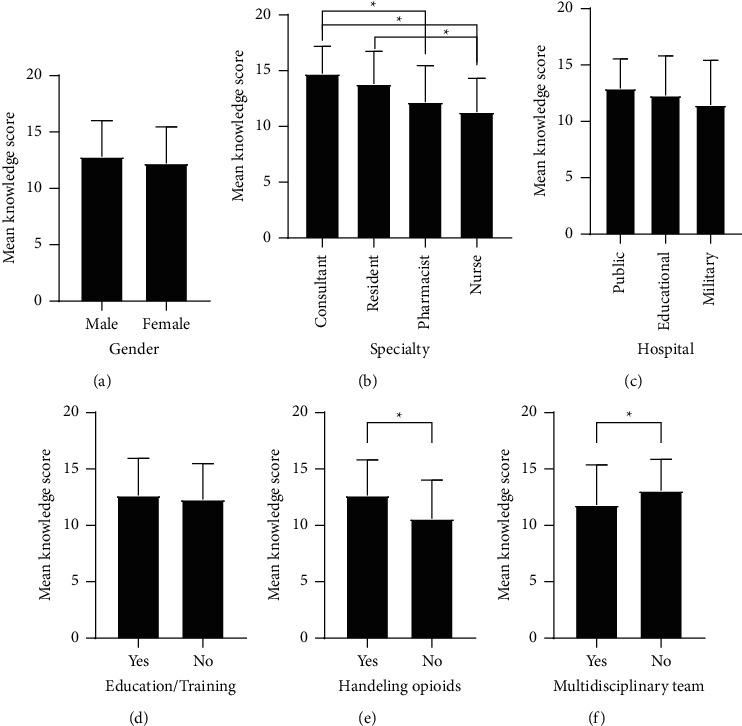
Knowledge of opioids based on demographic and practice characteristics of healthcare professionals. Scores of opioid knowledge were compared according to (a) gender, (b) specialty, (c) hospital, (d) prior education/training in pain management, (e) handling opioids, and (f) working in a multidisciplinary team. ^∗^ indicates a statistically significant difference at *p* < 0.05.

Figure 1 represents an analysis of the effect of selected demographic and practice characteristics on the knowledge of opioids among healthcare professionals. Mean knowledge scores were significantly different according to the specialty of healthcare professionals (*F* = 13.5, *p* < 0.001, Figure 1(b)). Knowledge scores were significantly higher for consultant doctors (mean = 14.7, SD = 2.5) compared to pharmacists (mean = 12.2, SD = 3.3) and nurses (mean = 11.3, SD = 3.01) (*p*=0.016 and *p* < 0.001, respectively). Further, scores were significantly higher for resident doctors (mean = 13.8, SD = 2.9) compared to nurses (*p* < 0.001). Besides, healthcare professionals who handled opioid analgesics (mean = 12.6, SD = 3.2) had significantly higher mean knowledge scores than those who did not handle opioids (mean = 10.6, SD = 3.4) (*t* = 2.549, *p*=0.012, Figure 1(e)). Interestingly, our results revealed that participants lacking multidisciplinary team management for cancer pain had significantly higher knowledge scores (mean = 13.1, SD = 2.8) than those who manage pain in a multidisciplinary team (mean = 11.8, SD = 3.6) (*t* = −2.8, *p*=0.005, Figure 1(f)). Gender, type of the hospital, and prior education/training in cancer pain management were not associated with significant differences in knowledge scores among healthcare professionals in this study (Figure 1). In addition, awareness of the WHO ladder and the number of patients requiring pain treatment did not influence the knowledge among respondents (data not shown). Pearson's correlation test revealed that knowledge scores were not correlated to the number of years of practice (*r* = 0.048, *p*=0.514).

Linear regression analysis revealed a three-variable model that is summarized in Table 3. The model highlighted that there is an association between the specialty of the respondent and the knowledge score (*p* < 0.001). As the respondent specialty changes from consultant physician to other specialties (resident doctor, pharmacist, and nurse), the mean knowledge score decreases by approximately one unit, while keeping other variables constant. In other words, there is strong evidence that being a consultant physician has an independent, statistically significant association with higher knowledge scores.

### 3.4. Perceived Barriers toward the Use of Opioids among Healthcare Professionals

Perceived barriers to the use of opioids for cancer pain management among healthcare professionals are listed in Table 4. “Fear of addiction by patients” was the most frequently perceived barrier by respondents (79.6%). Other highly recognized barriers were fear of adverse effects by patients (67.2%), lack of training programs on opioid dosing and monitoring (63.7%), limited education/training regarding prescribed opioids (55.2%), and insufficient knowledge of pain management protocols (53.7%). Barriers least perceived by healthcare professionals were the lack of adequate knowledge regarding opioid regulations (27.9%) and the time and effort spent in opioid inventory every shift (20.4%).

## 4. Discussion

Management of cancer pain has been a constant challenge to oncologists. Despite the ongoing efforts to advance pain management through numerous treatment guidelines and recommendations, undertreatment of cancer pain continues to be a global problem in which patients with cancer are inadequately treated for pain and are not receiving appropriate analgesia [19]. The WHO analgesic ladder, introduced in 1986, remains a highly recognized and useful tool for cancer pain management worldwide [13]. Based on the WHO recommendations, regularly dosed immediate-release or slow-release oral morphine should be considered to maintain pain relief in all patients with chronic cancer pain [13]. Besides, immediate-release oral morphine should be used as a rescue medicine for episodes of breakthrough pain [13, 16]. The WHO guidelines also recommend the use of nonopioid adjuvants in conjunction with opioids for cancer pain management such as steroids, anticonvulsants, antidepressants, and bisphosphonates [13]. Adjuvants have been found to improve response to opioids and provide better control of pain [30]. Nevertheless, adjuvants are currently underutilized in cancer pain management [13]. In addition, oral administration of opioids is usually preferable to avoid the discomfort, inconvenience, and expense of parenteral administration. In our study, 60.2% of healthcare professionals working at Oncology Units were aware of the WHO ladder for cancer pain treatment. Although this rate was similar to that in a study by Singh et al. in which 55% of medical practitioners reported knowledge of the WHO ladder, [21] these rates are considered suboptimal and awareness of the recommendations of the WHO ladder needs to be emphasized among healthcare professionals, especially those providing direct cancer care.

Opioids comprise a class of a large number of narcotics that include naturally derived alkaloids from the resin of the opium poppy as well as related synthetic and semisynthetic compounds, all of which are useful in clinical medicine [15, 31]. Opioids are also classified based on their interaction with opioid receptors into agonists, partial agonists, and antagonists [15]. All members of the opioid drug class share common pharmacological features as agonists of the MOR located along the nociceptive pathway to produce analgesic activity [15, 16, 31]. Morphine is considered the prototype opioid analgesic to which all other opioids are compared. Activation of MOR is also associated with adverse effects of opioids such as sedation, constipation, nausea, vomiting, and respiratory depression [15]. Upon the initiation of opioid analgesics, almost all patients will need regular laxative therapy for the prevention of constipation with senna and polyethylene glycol being the most commonly used [16]. Additionally, preventative antiemetics should be considered upon the initiation of opioid analgesics with metoclopramide being particularly useful for such an indication [16]. In this survey, healthcare professionals were mostly aware of the indication to use opioids for moderate-to-severe cancer pain, chronic pain, and breakthrough pain. Morphine and tramadol were the most handled opioids by participants in our study. These findings have been replicated in previous studies where most healthcare providers were aware that opioids are the mainstay analgesics to treat moderate-to-severe cancer pain [21]. Nevertheless, the knowledge on opioid pharmacology, administration, dosing, equianalgesic dosing, titration, rotation, and adverse effects was remarkably suboptimal in our study, a finding that was also reported by several other studies that described inadequate knowledge of opioid analgesics among healthcare professionals [18, 21, 29, 32–34]. In a study by Kim et al., the questions least answered correctly among Korean physicians were those regarding equianalgesic dosing for opioid rotation (12.8%) and the general principles of the adverse effects of opioids (47.2%) [18]. A survey of 219 physicians from Thailand showed that 62.1% of them had inadequate knowledge of opioids for cancer pain management [29]. Alternatively, a survey of medical oncologists in Spain revealed that many oncologists had good knowledge of the mechanism of action of opioids (79.5%) and good skills to manage opioid-related bowel dysfunction (76.7%) and opioid titration practices (87.7%) [26]. Studies also showed inadequate knowledge of cancer pain management among nurses in China, Iran, and United Arab Emirates [35–37]. A study on Jordanian nurses showed fair knowledge of cancer pain management with deficits in pharmacological management and negative attitudes toward opioid addiction and pain assessment [25].

Approximately, 80% of cancer patients will change the type of opioid drug at least once during cancer treatment [16]. Opioid rotation is mainly indicated in cases of opioid-induced neurotoxicity or inadequate analgesic activity. Our study showed that 59.7% of healthcare professionals were aware of this fact compared to 78.1% of physicians in Spain who would use opioid rotation in case of opioid-induced neurotoxicity [26]. Notably, our results showed that the majority of participants failed to identify the classic opioid overdose triad composed of pinpoint pupils (i.e., miosis), respiratory depression, and a decreased level of consciousness [38]. Though a rare adverse effect of opioids, particularly if administered at the appropriate dosage, respiratory depression could be life-threatening [16]. In our study, more than half of the respondents (58.7%) believed that respiratory depression is common even when opioids are administered at appropriate doses which implies false perceptions and negative views of these drugs, which could further hinder their use for cancer pain relief. Nevertheless, if severe respiratory depression or opioid overdose is suspected, a reversal of the effects of opioid analgesics is indicated. In such settings, the administration of naloxone is most appropriate to stop the effect of opioids by acting as an antagonist at all classical opioid receptors [15, 31]. Most respondents in our study were aware of the use of naloxone to stop opioid overdose (82.6%).

Opioids may cause euphoria and produce reward by acting on their target receptors, making them drugs of abuse [15, 16]. Addiction to opioids is perceived as a complex and advanced stage of substance use disorder associated with detrimental health and social consequences [31]. Chemical coping is defined as “the inappropriate and/or excessive use of opioids to cope with emotional distress rather than purely physical pain” [16, 39]. It implies the occasional overuse of opioids to cope with emotional distress without the craving, behavioral, and functional impairments that typically characterize addiction [39]. Addiction and chemical coping have been reported among cancer patients using opioids for cancer pain treatment [31]. A prospective study of advanced cancer patients revealed that 18% were diagnosed as chemically coping by palliative medicine specialists [40]. In our study, the majority of healthcare professionals (81.6%) falsely believed that opioid analgesics have a high risk of addiction, while paradoxically most of them were unable to accurately define addiction and chemical coping. Systematic reviews and meta-analyses demonstrated that the use of opioid analgesics for chronic pain management is associated with a low risk of iatrogenic addiction [41–43]. Our results are similar to findings by Singh et al. in which 45.3% of Indian medical practitioners were able to correctly define addiction [21]. Together, the high-perceived risk of addiction along with other negative views among our sample might explain, at least in part, the low prescribing rates of opioids to cancer patients in our settings and the constant high rates of undertreatment of cancer pain. Improving awareness of addiction and chemical coping among healthcare professionals is essential to recognize warning signs associated with opioid abuse and provide needed assistance to cancer patients in a proactive approach [39].

Different factors influenced the level of knowledge of healthcare professionals regarding opioids. Our results showed that medical doctors had greater knowledge compared to both pharmacists and nurses. This finding has been observed in earlier studies [17, 20, 44]. Although Jo et al. reported better knowledge of pain management among physicians than among nurses, both groups had inadequate knowledge regarding the pharmacology and adverse effects of opioids [20]. Our results indicated better knowledge of opioids among respondents who handled them; however, the level of knowledge was not influenced by previous education and/or training in cancer pain management or the knowledge of the WHO ladder. In contrary to our findings, education on pain management was associated with better knowledge among nurses [20, 25, 45]. Knowledge of the WHO three-step ladder was also associated with better knowledge of opioids for cancer pain management among physicians compared to those who lack knowledge of the WHO guidelines [29]. Interestingly, knowledge of opioids was higher among healthcare professionals who do not work among a multidisciplinary pain team in our study. An explanation of this may rely on the fact that lacking such a team necessitates a higher level of self-reading and learning among healthcare professionals who serve at Oncology Units and handle opioid analgesics. Interestingly, Ger et al. reported that physicians tended to have inadequate knowledge of opioid prescribing if they have the perception of good medical school training in cancer pain management [34]. Knowledge of opioids was not influenced by the number of years of experience for healthcare professionals in our study, a similar finding to a recent study by Su et al. showing that the duration of work experience did not affect cancer pain management for healthcare professionals in China [46].

Several barriers are known to attribute to inadequate management of cancer pain and are commonly categorized into three groups: healthcare professional-related, healthcare system-related, and patient-related [19]. Common professional-related barriers include poor assessment of pain, insufficient knowledge of cancer pain and management guidelines, the reluctance to prescribe opioids due to concerns of addiction risk and respiratory depression, and the low priority given to pain management in the cancer care plan [18, 19, 26]. System-related barriers included limited access to opioids, lacking the availability of different types of opioids, and lacking clear guidelines on cancer pain management [18, 19, 21]. Patient-related barriers included fear of adverse effects, fear of addiction to opioids, and not reporting pain. In our study, fear of addiction by patients was the most frequently reported barrier to opioid utilization for cancer pain management. Several previous studies reported that poor pain assessment was a major barrier to pain management [28, 34, 47]. In agreement with our results, patient reluctance to take opioids or report pain was a commonly reported barrier by healthcare professionals [28, 47]. Alternatively, reluctance to prescribe opioids by healthcare professionals was indicated by almost one-third of respondents in our study, while it was the least perceived barrier by physicians in China (21.0%) [28]. In our study, a lack of training programs on opioid dosing and monitoring was the most commonly reported barrier related to the healthcare system (63.7%). Lack of training opportunities and insufficient knowledge of cancer pain management were also barriers reported by physicians in previous studies [20, 29, 34]. In a recent Indian survey of medical practitioners, inadequate knowledge about opioid analgesics was the most indicated barrier (76.4%) [21]. Excessive regulation of opioid analgesics and fear of regulatory oversight were the least perceived healthcare system-related barriers by our respondents. However, these barriers were reported by medical oncologists and practitioners in USA and India [21, 47].

Almost half of the healthcare professionals (47.3%) in this study indicated the management of cancer pain by a multidisciplinary team in their Oncology Units. Compared to previous studies, this rate is relatively higher in which the involvement of a multidisciplinary team was indicated by a small proportion of medical oncologists in Spain (<10%) [26]. Interestingly, our study could be the first to assess the knowledge of pharmacists working at Oncology Units regarding opioids and cancer pain management. Recommendations to include pharmacists as members of palliative care teams who can take active roles in medical rounds were previously made by researchers [48]. Nevertheless, the role of pharmacists in cancer pain management is limited. A cross-sectional study by Nasser et al. reported that physicians in Lebanon were more likely to consult with another physician (65%) rather than a pharmacist (12%) when treating pain in patients [49]. In our survey, the knowledge scores of pharmacists were comparable to resident doctors but greater than nurses. Pharmacists could be particularly helpful when it comes to pharmacological treatments, opioid dosing, opioid equianalgesic dosing, titration, and management of adverse effects. Greater integration of pharmacists into multidisciplinary pain teams is expected to improve cancer pain management and opioid handling.

This survey has a few limitations. We conducted purposive sampling and the survey was limited to a small sample size of healthcare professionals. However, the high response rate and the inclusion of the three Oncology Units at Jordan would be helpful to reflect on the current practice settings and to understand the actual deficits and areas needing improvement in cancer pain management in Jordan.

## 5. Conclusions

Effective management of cancer pain requires multidisciplinary efforts from healthcare professionals providing cancer care. Our findings demonstrate the urgent need for developing innovative interventions to improve the knowledge of opioid analgesics and the understanding of and adherence to pain management guidelines among healthcare professionals in Jordan. Educational interventions for healthcare students at the undergraduate and graduate levels are mandatory. These interventions may call for the integration of cancer pain management into the existing course work or establish new modules that focus on pain and palliative care in cancer patients. This should go along with establishing continuous education programs for professional healthcare workers on the levels of their associations and institutions to maintain and update their workers with knowledge of pain management and opioid analgesics. The activation for the role of pharmacists might contribute positively to improved pain control in cancer patients. Education of patients is also necessary to reduce the negatively perceived barriers about opioids. Institutions should also facilitate the use of opioid analgesics by relieving extensive regulatory barriers.

## Figures and Tables

**Table 1:** Demographics and practice characteristics.

Characteristic	*n* (%)
Gender	
Male	87 (43.3)
Female	114 (56.7)
Speciality	
Consultant doctor	23 (11.4)
Resident doctor	53 (26.4)
Pharmacist	26 (12.9)
Nurse	99 (49.3)
Hospital	
Public	87 (43.3)
Educational	89 (44.3)
Military	25 (12.4)
Education/training in cancer pain management	
Yes	99 (49.3)
No	102 (50.7)
As an estimate from your practice setting, what is the percentage of cancer patients suffering pain you encounter per month?	
≤25%	18 (9.0)
26–50%	37 (18.4)
51–75%	61 (30.3)
>75%	85 (42.3)
As an estimate from your practice setting, what is the average number of cancer patients who required pain management per month?	
None	3 (1.5)
1–4	18 (9.0)
5–10	40 (19.9)
>10	140 (69.7)
Cancer pain is managed by a multidisciplinary team in my hospital/unit	
Yes	95 (47.3)
No	106 (52.7)
Specific protocols are used for cancer pain management in my hospital/unit	
Yes	74 (36.8)
No	105 (52.2)
I do not know	22 (10.9)
I am aware of the World Health Organization (WHO) pain ladder	
Yes	121 (60.2)
No	80 (39.8)
Have you ever handled opioids to manage pain in a cancer patient?	
Yes	184 (91.5)
No	17 (8.5)
Type of opioid	
Morphine	188 (93.5)
Tramadol	184 (91.5)
Fentanyl	123 (61.2)
Pethidine (meperidine)	66 (32.8)
Codeine	40 (19.9)
Oxycodone	28 (13.9)
Methadone	19 (9.5)
Others	3 (1.5)
Are you aware of adjuvant medicines for cancer pain management?	
Yes	178 (88.6)
No	23 (11.4)
Type of adjuvant	
Steroids	160 (79.6)
Anticonvulsants	116 (57.7)
Antidepressants	99 (49.3)
Bisphosphonates	38 (18.9)
Others	3 (1.5)

Other opioids included hydrocodone and hydromorphone. Other adjuvants included paracetamol.

**Table 2:** Knowledge regarding opioids and their use for cancer pain management among healthcare professionals.

No.	Question/item	Answer	True	False	I do not know
1.	Opioids should be prescribed to patients suffering from moderate-to-severe cancer pain	True	183 (91.0)	12 (6.0)	6 (3.0)
2.	Opioids should be only used when nonopioid analgesics (paracetamol, NSAIDs) are not effective to manage cancer pain	False	104 (51.7)	91 (45.3)	6 (3.0)
3.	Any opioid may be considered for maintenance of pain relief (alone or in combination with NSAIDs and/or paracetamol)	True	109 (54.2)	72 (35.8)	20 (10.0)
4.	All patients with chronic cancer pain should be started on regular opioids, ideally using an extended-release formulation	True	141 (70.1)	45 (22.4)	15 (7.5)
5.	Breakthrough cancer pain should be treated with a rescue medicine such as morphine in its immediate-release formulation	True	164 (81.6)	16 (8.0)	21 (10.4)
6.	Opioid analgesics work mainly by binding the mu-opioid receptors located along the nociceptive pathway	True	79 (39.3)	20 (10.0)	102 (50.7)
7.	Parenteral administration of opioids is preferred to oral administration in cancer pain management	False	84 (41.8)	95 (47.3)	22 (10.9)
8.	Morphine does not have a ceiling effect (no maximum dosage limit)	True	120 (59.7)	56 (27.9)	25 (12.4)
9.	When switching from injected morphine to oral morphine the dose should be reduced by half	False	75 (37.3)	66 (32.8)	60 (29.9)
10.	The starting dose for opioid analgesics is equivalent to 30 mg of morphine per day orally	True	67 (33.3)	71 (35.3)	63 (31.3)
11.	In opioid titration, the minimal clinically important increase or decrease in dose is approximately 30% of the daily dose	True	66 (32.8)	37 (18.4)	98 (48.8)
12.	Opioid titration is conducted as a percentage rather than an absolute number	True	94 (46.8)	21 (10.4)	86 (42.8)
13.	Upon cessation of opioid therapy, the opioid dose must be tapered by 10% per week in cancer patients with long-term use	True	83 (41.3)	22 (10.9)	96 (47.8)
14.	Laxatives should be started together with opioids	True	154 (76.6)	39 (19.4)	8 (4.0)
15.	Opioid rotation is indicated to manage opioid-induced neurotoxicity	True	120 (59.7)	41 (20.4)	40 (19.9)
16.	Opioid analgesics have a high risk of addiction	False	164 (81.6)	34 (16.9)	3 (1.5)
17.	Opioid-induced respiratory depression is common even when opioids are given at appropriate doses	False	118 (58.7)	74 (36.8)	9 (4.5)
18.	The use of adjuvants is only indicated when patients are using opioids	False	66 (32.8)	106 (52.7)	29 (14.4)
19.	Seizures, dilated pupils, and bradyarrhythmia are the classic triad for opioid overdose	False	136 (67.7)	45 (22.4)	20 (10.0)
20.	If opioid overdose is suspected, the best immediate response is the administration of naloxone	True	166 (82.6)	23 (11.4)	12 (6.0)
21.	The need to progressively increase the opioid dose to maintain the same level of analgesia is known as:	Tolerance	Correct responses: 157 (78.1)		
22.	Occurrence of withdrawal symptoms following abrupt reduction of the opioid dose is known as:	Dependence	Correct responses: 119 (59.2)		
23.	Impaired control over opioid use, craving, compulsive, and continued use despite harm is known as:	Addiction	Correct responses: 94 (46.8)		
24.	The use of opioids by patients to manage emotional distress rather than purely physical pain is known as:	Chemical coping	Correct responses: 80 (39.8)		

Data are presented as *n* (%).

**Table 3:** Linear regression analysis for factors associated with knowledge of opioids among healthcare professionals.

Variable	B	Std. error	*p* value	95% confidence interval	
				Lower bound	Upper bound
(Constant)	14.387	1.097	<0.001^*∗*^	12.224	16.549
Specialty	−1.066	0.267	<0.001^*∗*^	−1.593	−0.539
Cancer pain is managed by a multidisciplinary team in my hospital/unit	−0.702	0.446	0.118	−1.582	0.179
Have you ever handled opioids to manage pain in a cancer patient?	1.342	0.782	0.088	−0.2	2.883

^∗^ indicates a statistically significant difference at *p* < 0.05.

**Table 4:** Barriers to the use of opioids in cancer pain management among healthcare professionals.

Barrier	*n* (%)
Barriers related to healthcare professionals	
Limited education/training regarding prescribed opioids	111 (55.2)
Insufficient knowledge of pain management protocols	108 (53.7)
Concerns about adverse effects of opioids by HCPs	104 (51.7)
Fear of drug addiction and dependence by HCPs	102 (50.7)
Inadequate experience in pain control	98 (48.8)
Inadequate pain assessment by HCPs	94 (46.8)
Reluctance to prescribe/administer opioids by HCPs	67 (33.3)
Lack of adequate knowledge regarding opioid regulations	56 (27.9)
Barriers related to healthcare system	
Lack of specialized training programs on opioid dosages and monitoring	128 (63.7)
Lack of multidisciplinary team that can aid in pain management decisions	96 (47.8)
Lack of access to a wide range of opioids	94 (46.8)
Limited stocks of different types of opioids in the hospital pharmacy	80 (39.8)
Lack of access to a wide range of analgesics	79 (39.3)
Excessive regulation of opioid drugs	56 (27.9)
Fear of regulatory oversight	49 (24.4)
Time and effort spent in opioid inventory every shift	41 (20.4)
Barriers related to patients	
Fear of addiction by patients	160 (79.6)
Fear of adverse effects by patients	135 (67.2)
Patient reluctance to take opioids	105 (52.5)
Inadequate pain reporting by patients	102 (50.7)
Fear of stigma related to opioid use	75 (37.3)

HCPs, healthcare professionals.

## Data Availability

The data used to support the findings of this study can be available from the corresponding author upon reasonable request.
